# Supplementary material for: COZOID: contact zone identifier for visual analysis of protein-protein interactions
Source: BMC Bioinformatics. 2018 Apr 6;19:125. doi: 10.1186/s12859-018-2113-6 (PMC5889581; doi:10.1186/s12859-018-2113-6)
Supplement: Supplementary file 2 — Software build. Executable binary file of the software tool. (ZIP 118,784 kb) [file 12859_2018_2113_MOESM2_ESM.zip › cozoid/java/docs/beansbinding-1.2.1-doc.zip!/org/jdesktop/beansbinding/PropertyStateEvent.html]

PropertyStateEvent (Beans Binding)


---


|  |  |  |
| --- | --- | --- |
| **Overview** \| **Package** \| **Class** \| **Use** \| **Tree** \| **Deprecated** \| **Index** \| **Help** | |  |
| **PREV CLASS**   **NEXT CLASS** | **FRAMES**    **NO FRAMES**     **All Classes** |  |
| SUMMARY: NESTED \| FIELD \| CONSTR \| METHOD | DETAIL: FIELD \| CONSTR \| METHOD |  |


---


## org.jdesktop.beansbinding Class PropertyStateEvent

```
java.lang.Object
  java.util.EventObject
      org.jdesktop.beansbinding.PropertyStateEvent
```

**All Implemented Interfaces:**: java.io.Serializable

---

``` public class PropertyStateEvent extends java.util.EventObject ```

An event characterizing a change in a `Property's` state for
a particular source object.

**See Also:**: `Property`, `PropertyStateListener`, Serialized Form

---

| **Field Summary** | |
| --- | --- |
| `static java.lang.Object` | `UNREADABLE`             Used to indicate that a particular value is unreadable. |

**Fields inherited from class java.util.EventObject**

`source`


**Constructor Summary**

`PropertyStateEvent(Property sourceProperty,
java.lang.Object sourceObject,
boolean valueChanged,
java.lang.Object oldValue,
java.lang.Object newValue,
boolean writeableChanged,
boolean isWriteable)`
  
          Creates an instance of `PropertyStateEvent` characterizing a
change in a `Property's` state for a particular source object.


| **Method Summary** | |
| --- | --- |
| `java.lang.Object` | `getNewValue()`             Returns the new value of the `Property` for the source object, or `UNREADABLE` if the `Property` is not currently readable for the source object. |
| `java.lang.Object` | `getOldValue()`             Returns the old value of the `Property` for the source object, or `UNREADABLE` if the `Property` was not previously readable for the source object. |
| `boolean` | `getReadableChanged()`             Returns whether or not the `Property's` readability has changed for the source object. |
| `java.lang.Object` | `getSource()`             Returns the `Property` whose state has changed. |
| `java.lang.Object` | `getSourceObject()`             Returns the source object for which the `Property's` state has changed. |
| `Property` | `getSourceProperty()`             Returns the `Property` whose state has changed. |
| `boolean` | `getValueChanged()`             Returns whether or not the `Property's` value has changed for the source object. |
| `boolean` | `getWriteableChanged()`             Returns whether or not the `Property's` writeability has changed for the source object. |
| `boolean` | `isReadable()`             Returns whether or not the `Property` is currently readable for the source object. |
| `boolean` | `isWriteable()`             Returns whether or not the `Property` is currently writeable for the source object. |
| `java.lang.String` | `toString()`             Returns a string representation of the `PropertyStateEvent`. |

**Methods inherited from class java.lang.Object**

`clone, equals, finalize, getClass, hashCode, notify, notifyAll, wait, wait, wait`

**Field Detail**

### UNREADABLE

```
public static final java.lang.Object UNREADABLE
```

:   Used to indicate that a particular value is unreadable.


**Constructor Detail**

### PropertyStateEvent

```
public PropertyStateEvent(Property sourceProperty,
                          java.lang.Object sourceObject,
                          boolean valueChanged,
                          java.lang.Object oldValue,
                          java.lang.Object newValue,
                          boolean writeableChanged,
                          boolean isWriteable)
```

:   Creates an instance of `PropertyStateEvent` characterizing a
    change in a `Property's` state for a particular source object.

    Note: To indicate a change in readability, specify `valueChanged`
    as `true` and reflect the readability status in the `oldValue`
    and `newValue` arguments.

    **Parameters:**: `sourceProperty` - the `Property` whose state has changed: `sourceObject` - the source object for which the `Property's` state has changed: `valueChanged` - whether or not the `Property's` value has changed for the source object: `oldValue` - the old value of the `Property` for the source object, or `UNREADABLE` if the `Property` was not previously readable for the source object: `newValue` - the new value of the `Property` for the source object, or `UNREADABLE` if the `Property` is not currently readable for the source object: `writeableChanged` - whether or not the `Property's` writeability has changed for the source object: `isWriteable` - whether or not the `Property` is now writeable for the source object **Throws:**: `java.lang.IllegalArgumentException` - if neither the value or the writeability has changed: `java.lang.IllegalArgumentException` - if `valueChanged` is `true` and both `oldValue` and `newValue` are `UNREADABLE`


**Method Detail**

### getSource

```
public final java.lang.Object getSource()
```

:   Returns the `Property` whose state has changed.
    The preferred way to access this value is via the
    `getSourceProperty()` method.

    :   **Overrides:**: `getSource` in class `java.util.EventObject`
    :   **Returns:**: the `Property` whose state has changed.

---


### getSourceProperty

```
public final Property getSourceProperty()
```

:   Returns the `Property` whose state has changed.

    :   **Returns:**: the `Property` whose state has changed.

---


### getSourceObject

```
public final java.lang.Object getSourceObject()
```

:   Returns the source object for which the `Property's` state has changed.

    :   **Returns:**: the source object for which the `Property's` state has changed

---


### getValueChanged

```
public final boolean getValueChanged()
```

:   Returns whether or not the `Property's` value has changed for the source object.

    :   **Returns:**: whether or not the `Property's` value has changed for the source object.

---


### getOldValue

```
public final java.lang.Object getOldValue()
```

:   Returns the old value of the `Property` for the source object,
    or `UNREADABLE` if the `Property` was not previously readable for the
    source object.

    Note: This method must only be called if `getValueChanged` returns
    `true`.

    :   **Returns:**: the old value of the `Property` for the source object or `UNREADABLE` **Throws:**: `java.lang.UnsupportedOperationException` - if the value hasn't changed

---


### getNewValue

```
public final java.lang.Object getNewValue()
```

:   Returns the new value of the `Property` for the source object,
    or `UNREADABLE` if the `Property` is not currently readable for the
    source object.

    Note: This method must only be called if `getValueChanged` returns
    `true`.

    :   **Returns:**: the new value of the `Property` for the source object or `UNREADABLE` **Throws:**: `java.lang.UnsupportedOperationException` - if the value hasn't changed

---


### getReadableChanged

```
public final boolean getReadableChanged()
```

:   Returns whether or not the `Property's` readability has changed for
    the source object. In particuler, this returns `true` if the value
    has changed and either the old value or new value is `UNREADABLE`,
    and `false` otherwise.

    :   **Returns:**: whether or not the `Property's` readability has changed for the source object.

---


### isReadable

```
public final boolean isReadable()
```

:   Returns whether or not the `Property` is currently readable for
    the source object. In particular, this returns `true` if and only
    if the new value is not `UNREADABLE`.

    Note: This method must only be called if `getReadableChanged` returns
    `true`.

    :   **Returns:**: whether or not the `Property` is currently readable for the source object. **Throws:**: `java.lang.UnsupportedOperationException` - if the readability hasn't changed

---


### getWriteableChanged

```
public final boolean getWriteableChanged()
```

:   Returns whether or not the `Property's` writeability has changed for
    the source object.

    :   **Returns:**: whether or not the `Property's` writeability has changed for the source object.

---


### isWriteable

```
public final boolean isWriteable()
```

:   Returns whether or not the `Property` is currently writeable for
    the source object.

    Note: This method must only be called if `getWriteableChanged` returns
    `true`.

    :   **Returns:**: whether or not the `Property` is currently writeable for the source object. **Throws:**: `java.lang.UnsupportedOperationException` - if the writeability hasn't changed

---


### toString

```
public java.lang.String toString()
```

:   Returns a string representation of the `PropertyStateEvent`. This
    method is intended to be used for debugging purposes only, and
    the content and format of the returned string may vary between
    implementations. The returned string may be empty but may not
    be `null`.

    :   **Overrides:**: `toString` in class `java.util.EventObject`
    :   **Returns:**: a string representation of this `PropertyStateEvent`


---


|  |  |  |
| --- | --- | --- |
| **Overview** \| **Package** \| **Class** \| **Use** \| **Tree** \| **Deprecated** \| **Index** \| **Help** | |  |
| **PREV CLASS**   **NEXT CLASS** | **FRAMES**    **NO FRAMES**     **All Classes** |  |
| SUMMARY: NESTED \| FIELD \| CONSTR \| METHOD | DETAIL: FIELD \| CONSTR \| METHOD |  |


---
